# An Accurate Millimeter-Wave Imaging Algorithm for Close-Range Monostatic System

**DOI:** 10.3390/s23104577

**Published:** 2023-05-09

**Authors:** Xinyi Nie, Chuan Lin, Yang Meng, Anyong Qing, Jan K. Sykulski, Ian D. Robertson

**Affiliations:** 1 School of Electrical Engineering, Southwest Jiaotong University, Chengdu 611756, China; 2 School of Electronic and Electrical Engineering, University of Leeds, Leeds LS2 9JT, UK; 3 School of Optoelectronic Engineering, Chongqing University of Posts and Telecommunications, Chongqing 400065, China; 4 School of Electronics and Computer Science, University of Southampton, Southampton SO17 1BJ, UK

**Keywords:** concealed weapon detection, Fourier transform technique, method of stationary phase, microwave imaging, national security

## Abstract

An efficient and more accurate millimeter-wave imaging algorithm, applied to a close-range monostatic personnel screening system, with consideration of dual path propagation loss, is presented in this paper. The algorithm is developed in accordance with a more rigorous physical model for the monostatic system. The physical model treats incident waves and scattered waves as spherical waves with a more rigorous amplitude term as per electromagnetic theory. As a result, the proposed method can achieve a better focusing effect for multiple targets in different range planes. Since the mathematical methods in classical algorithms, such as spherical wave decomposition and Weyl identity, cannot handle the corresponding mathematical model, the proposed algorithm is derived through the method of stationary phase (MSP). The algorithm has been validated by numerical simulations and laboratory experiments. Good performance in terms of computational efficiency and accuracy has been observed. The synthetic reconstruction results show that the proposed algorithm has significant advantages compared with the classical algorithms, and the reconstruction by using full-wave data generated by FEKO further verifies the validity of the proposed algorithm. Finally, the proposed algorithm performs as expected over real data acquired by our laboratory prototype.

## 1. Introduction

Terrorist attacks around the world are still at a high level because regional and religious conflicts have been intensifying [1]. Crowded places such as transportation hubs (airports, railway stations, metro stations, etc.), entertainment venues, government agencies, and schools are primary targets. These threats can result in not only loss of life and property, but also social anxiety and panic. Therefore, it is extremely urgent and challenging to ensure public security. It is critical that prohibited items such as weapons and explosives are detected before they enter those crowded public areas.

Conventional approaches, including metal detectors, infrared detectors, and X-ray systems [2], already exist. To some extent, these approaches are effective but suffer some drawbacks in practical applications. Metal detectors can only detect metal targets. Meanwhile, many modern hazardous articles are now made using advanced technologies, such as plastic, ceramics (e.g., knives), and liquid explosives, that cannot be detected by using metal detectors. Infrared detectors can realize imaging but are seriously affected by the environment, and the image quality is inherently not high [3]. X-ray systems have remarkable performance in terms of penetrability and image quality, so they are a good detection method for carry-on luggage. However, even low-dose X-ray cannot be accepted as a means of personnel security inspection, since X-rays are ionizing radiation and have a cumulative effect.

Millimeter waves are high-frequency electromagnetic waves with relatively short wavelengths in the 1–10 mm range and offer good penetrability. They are capable of providing good spatial resolution and penetrating common clothing and packaging materials. Moreover, millimeter waves are nonionizing. Thus, a millimeter-wave system is well suited for personnel security screening.

In terms of imaging techniques, the back-projection (BP) algorithm is one of the most accurate methods in the spatial domain [2,4,5,6,7,8,9]. Boag [8,9] attempted to speed up the BP algorithm by using multilevel domain decomposition. Unfortunately, the demonstrated acceleration was achieved at the cost of accuracy. As a matter of fact, at acceptable accuracy for engineering practice, the demonstrated efficiency may even be worse than that of the original BP algorithm. Therefore, its heavy computational burden is still a severe issue, and it is impractical for real-time security checks. It is noted that the scattering data are the convolution of reflectivity function and Green’s function in spatial domain, which can be more efficiently handled as the multiplication of their Fourier transforms in the wavenumber domain and converted back through FFT technique. Therefore, many efficient wavenumber-domain algorithms have been developed, such as the holographic algorithm [10,11,12,13,14,15,16,17,18,19,20,21,22,23,24,25,26,27,28,29,30], range Doppler (RD) algorithm [31,32], chirp scaling (CS) algorithm [33,34], and range migration algorithm (RMA) [35,36,37], among which the holographic algorithm has been being heavily studied for its proven commercial potential. In particular, monostatic configuration is often assumed for simplicity and efficiency. In this paper, this practice is also adopted.

Close-range imaging systems based on a monostatic configuration have been studied in many works [7,11,13,16,17,18,19,20,21,22,23,24,25,26,27,28,29,32,36,38]. Most of the studies neglected the amplitude terms of both incident and scattered spherical waves for simplicity [11,16,17,18,19,20,21,22,23,32,36]. Such negligence might result in some severe issues because the propagation loss is very important while the undulate scale of objects in security screening is comparable to the range of distances in close-range millimeter-wave imaging [10,11,35,39,40].

A recent remedy partly took propagation loss into account [25,26]. The scattered signal is treated as a spherical wave, which is closer to the actual physical model. Therefore, the quality of the reconstruction image can be improved to some extent. Nevertheless, the physical model is still not consistent with the actual physical model.

To eradicate the problem, the dual path propagation loss has to be adequately taken into account [4,7,38]. It is straightforward for BP algorithm to compensate the dual path propagation loss by directly including the factor *R*^2^ in its formulation [4,7]. However, the computation burden is too heavy. A similar computationally inefficient approach is observed in the range stacking algorithm, where the scattering data were transformed into the slant range spatial domain and then multiplied by an amplitude factor *R*^2^ [38]. Surprisingly, a factor *R*^4^, inconsistent with electromagnetic theory, appeared in [27].

In research to date, the dual path propagation loss, $1/R^{2}$, has hardly been considered in monostatic holographic algorithms. A close look at the mathematical formulation reveals that the mathematical methods in holographic algorithms, such as spherical wave decomposition [11] and Weyl identity [25], stop working if the term $1/R^{2}$ is involved. The method of stationary phase (MSP) is therefore chosen to overcome this difficulty. A holographic reconstruction algorithm for a monostatic system with full consideration of dual path propagation loss for close-range millimeter-wave imaging is accordingly proposed in this paper.

The proposed method treats incident waves and scattered waves as spherical waves with more rigorous amplitude terms as per electromagnetic theory. That is to say, the propagation loss is more accurately taken into account. Thus, the physical method is more consistent with the actual scenario. Consequently, it can achieve a better focusing effect for multiple targets in different range planes. The proposed algorithm is derived through MSP. Meanwhile, FFT is also employed to transform spatial domain scattering data to the wavenumber domain for efficient imaging. The proposed algorithm has been validated by improved reconstructed images.

It has to be pointed out that the dual path propagation loss in MIMO and bistatic systems has already been addressed [35,41,42,43]. However, as far as the authors know, our imaging formula is by no means a simple degeneration but a brand-new formulation. More importantly, our algorithm significantly outperforms those degenerated algorithms when the focusing effect plays a role. Due to page limitations, details will be published elsewhere separately [44].

## 2. Theory

**Figure 1:**
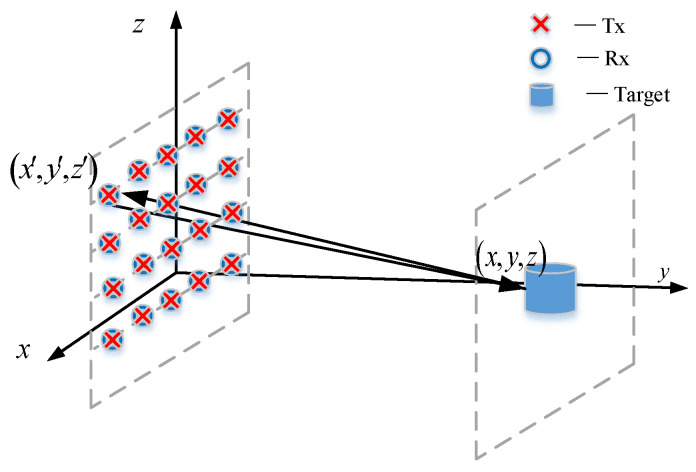
The geometry of imaging system.

The geometry of the imaging system is shown in Figure 1. The transmitting and receiving antennas are assumed to be positioned at (*x*′, *y*′, *z*′) on the plane of *y = y*′. An arbitrary point on the target is represented as (*x*, *y*, *z*) with reflectivity function *f*(*x*, *y*, *z*).

In essence, a transmitter emanates a spherical wave, which will illuminate the object and be reflected by the object. Each point on the object can be regarded as a secondary source transmitting spherical waves. Consequently, the echo signal measured by the corresponding receiver would be the superposition of all the spherical waves emanating from all the points on the object.

Supposing that the scattering process satisfies the Born approximation according to electromagnetic theory [13,45], the measured backscattered data is

(1) $$s\left(x^{\prime },z^{\prime },k\right)=\int \int \int_{V}\;f\left(x,y,z\right)\frac{\exp\left(-j2kR\right)}{{\left(4\pi R\right)}^{2}}dxdydz$$

where $j=\sqrt{-1}$, k=2π/λ is the spatial wave number, and λ is the wavelength. The term $\exp\left(-j2kR\right)/R^{2}$ stands for the strict spherical wave expression containing the incident wave and scattered wave. It is noted that the constant term, $1/{\left(4\pi \right)}^{2}$, will be omitted for convenience since the constant term has no effect on imaging results. The distance from the transceiver to the object is

(2) $$R=\sqrt{{\left(x-x^{\prime }\right)}^{2}+{\left(y-y^{\prime }\right)}^{2}+{\left(z-z^{\prime }\right)}^{2}}$$

The Fourier transform of the measured backscatter data $s\left(x^{\prime },z^{\prime },k\right)$ can be expressed as

(3) $$S\left(k_{x},k_{z},k\right)=\int_{-\infty }^{\infty }\int_{-\infty }^{\infty }s\left(x^{\prime },z^{\prime },k\right)e^{-jk_{x}x^{\prime }}e^{-jk_{z}z^{\prime }}dx^{\prime }dz^{\prime }$$

Substituting (1) into (3) yields

(4) $$\begin{array}{ll} S\left(k_{x},k_{z},k\right)= & \int \int \int_{V}f\left(x,y,z\right)\\ & \;\;\;\cdot \int_{-\infty }^{\infty }\int_{-\infty }^{\infty }\frac{\exp\left(-j2kR\right)}{R^{2}}e^{-jk_{x}x^{\prime }}e^{-jk_{z}z^{\prime }}dxdydzdx^{\prime }dz^{\prime } \end{array}$$

For the sake of simplicity, the double integral about $x^{\prime }$ and $z^{\prime }$ in (4) can be represented as

(5) $$E\left(k_{x},k_{z},k\right)=\int_{-\infty }^{\infty }\int_{-\infty }^{\infty }\frac{\exp\left(-j2kR\right)}{R^{2}}e^{-jk_{x}x^{\prime }}e^{-jk_{z}z^{\prime }}dx^{\prime }dz^{\prime }$$

The asymptotic expansion of the integral of (5) can be obtained by the MSP as [36,41,45]

(6) $$E\left(k_{x},k_{z},k\right)=\frac{j\pi }{k}\frac{1}{y-y^{\prime }}e^{-j\left(\sqrt{{\left(2k\right)}^{2}-k_{x}^{2}-k_{z}^{2}}\left(y-y^{\prime }\right)+k_{x}x+k_{z}z\right)}$$

Please see the Appendix A for more details. Substituting (6) into (4), we have

(7) $$\begin{array}{ll} S\left(k_{x},k_{z},k\right)= & \int \int \int_{V}\frac{1}{y-y^{\prime }}f\left(x,y,z\right)\\ & \;\;\cdot \frac{j\pi }{k}e^{-j\left(\sqrt{{\left(2k\right)}^{2}-k_{x}^{2}-k_{z}^{2}}\left(y-y^{\prime }\right)+k_{x}x+k_{z}z\right)}dxdydz \end{array}$$

Let

(8) $$k_{y}=\sqrt{{\left(2k\right)}^{2}-k_{x}^{2}-k_{z}^{2}}$$

Then, (7) can be re-formulated as

(9) $$\begin{array}{l} \frac{k}{j\pi }S\left(k_{x},k_{z},k\right)e^{-jk_{y}y^{\prime }}\\ \;=\int \int \int_{V}\frac{f\left(x,y,z\right)}{y-y^{\prime }}e^{-j\left(k_{x}x+k_{y}y+k_{z}z\right)}dxdydz≜F\left(k_{x},k_{y},k_{z}\right) \end{array}$$

where $F\left(k_{x},k_{y},k_{z}\right)$ is the Fourier transform of $f\left(x,y,z\right)/\left(y-y^{\prime }\right)$.

Thus, the object can be reconstructed as

(10) $$\begin{array}{l} f\left(x,y,z\right)=\frac{y-y^{\prime }}{j\pi }\\ \;\cdot {\mathrm{FT}}_{3D}^{-1}\left\{{\left[k\cdot {\mathrm{FT}}_{2D}{\left.\left[s\left(x^{\prime },z^{\prime },k\right)\right]\right|}_{x^{\prime },z^{\prime }}e^{-jk_{y}y^{\prime }}\right]}_{\mathrm{Interpolation}}\right\} \end{array}$$

where FT_2D_ represents the 2-D Fourier transform and ${\mathrm{FT}}_{3D}^{-1}$ represents the 3-D inverse Fourier transform. It should be noted that $y^{\prime }$ on the right-hand side of (10) is the transceiver plane. Therefore, it is accurately known in both measurement of backscattered data and device under test (DUT) profile reconstruction once the geometry of the imaging system is fixed.

The proposed method takes into account the propagation loss of the spherical wave in free space. Therefore, the proposed algorithm is based on a more precise physical model, which will bring an improvement in the reconstruction results. It should also be pointed out that (10) will be close to the traditional method if the DUT plane is parallel to the image plane. However, a parallel DUT plane is ideal, so it is hardly possible for practical personnel screening. Additionally, a DUT is intermediate rather than far in practical personnel screening. Our algorithm will keep its advantage in practical personnel screening when the wavy DUT distance plays an important role.

## 3. Results and Discussions

Numerical simulations and experiments are used to test the performance of the proposed algorithm. Different kinds of test scenarios are purposely employed. A point target simulation is used to test resolution. Three rectangle objects are used to test the performance of the algorithm. The capability of 3-D imaging is presented by 3-D full-wave data. Finally, reconstruction with real data also validates the performance.

In order to verify the improvement of our proposed method, Sheen’s method [11] and Meng’s method [25] are chosen as competitors. All the algorithms are realized with self-developed MATLAB codes. The computational platform is a desktop computer with a regular Intel 64-bit 3.19-GHz CPU and 32 GB RAM.

In the numerical experiments, the simulated scattered data were obtained through (1). The system parameters used to generate the synthetic scattered data are presented in Table 1

### 3.1. Point Spread Function

The point spread function (PSF) quantifies the capability of a system to image an arbitrary point scatterer. Meanwhile, it can also be considered as an essential approach to evaluate imaging algorithms, since point scatterer simulations provide distinctive insight into other aspects of image quality unavailable by realistic target.

**Figure 2:**
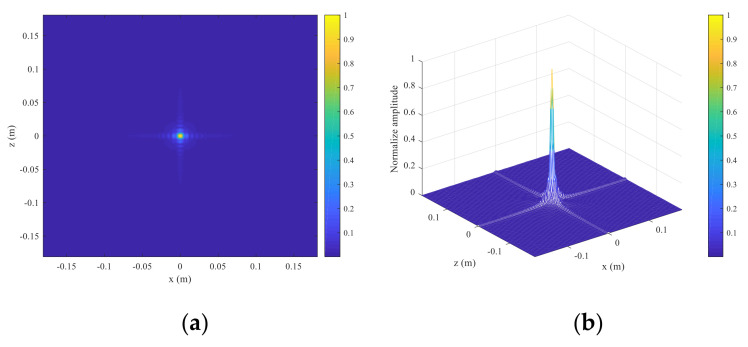
The reconstruction results of point scatterer in scale normalized to the maximum. (**a**) The reflectivity contour projected to *xz* plane. (**b**) The 3-D view of the reflectivity projected to the *xz* plane.

A point scatterer is placed at (0 m, 0.4 m, 0 m) in front of the transceiver plane. The transceiver plane is located at *y =* 0. In order to better address the point object, the equidistant sample is 2 mm instead of 5 mm, which strictly satisfies the Nyquist sampling theorems. Figure 2 shows the reconstructed PSF projected to the *xz* plane. It can be seen that the energy of the scattered field is mainly focused on (0 m, 0 m) and that the spreading phenomenon occurs.

**Figure 3:**
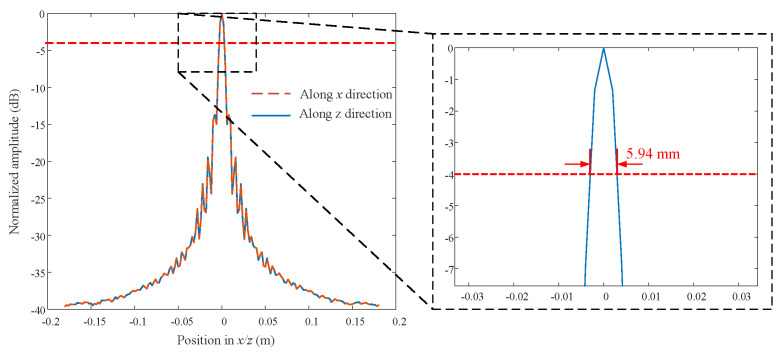
Profiles along *x*(*z*) in dB scale normalized to the maximum.

**Figure 4:**
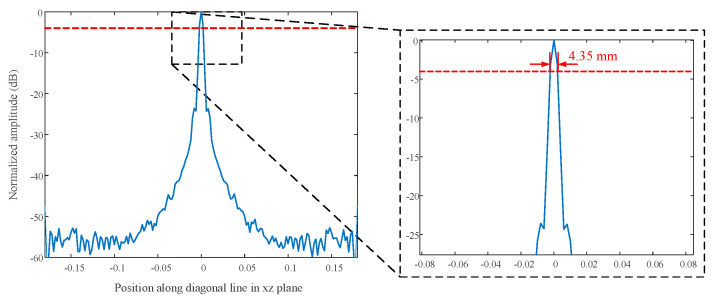
Profiles along *x* = *z* in dB scale normalized to the maximum.

**Figure 5:**
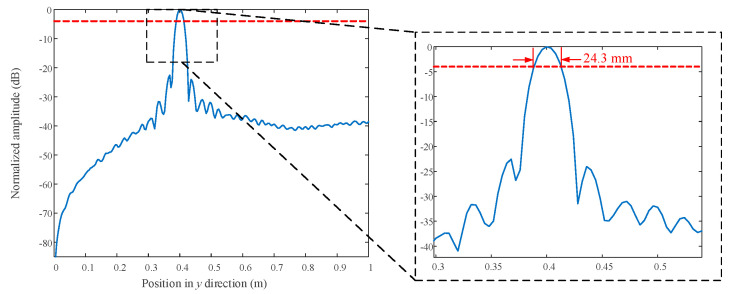
Profiles along *y* in dB scale normalized to the maximum.

The normalized PSF along the *x* and *z* direction in the *y* = 0.4 m plane is shown in Figure 3. The width of both of the amplitude lines at −4 dB [46,47] is 5.94 mm. The normalized PSF along *x* = *z* in the *y* = 0.4 m plane is shown in Figure 4. The width of the amplitude line at −4 dB is 4.35 mm. The PSF along *y* is shown in Figure 5. The width of the amplitude line at −4 dB is 24.3 mm. The resolution of the PSF is thus 5.94 mm along *x* and *z*, 4.35 mm along the diagonal at 45° relative to *x*, and 24.3 mm in the range direction. Theoretically, the lateral resolution and range resolutions are about $\lambda_{c}/2\approx$ 5 mm and $c/\left(2B\right)\approx$ 25.8 mm [35,36,48]. Generally speaking, the numerical resolutions from PSF are consistent with the theoretical ones.

### 3.2. The Synthetic Data

**Figure 6:**
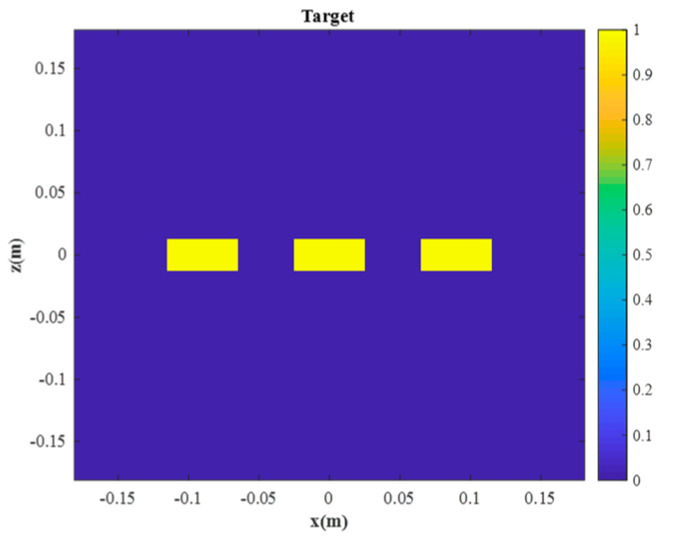
The 2-D object under test.

Three identical rectangular objects, which are considered the objects under test, are illustrated in Figure 6. The length and width of the rectangular objects are 24 mm and 48 mm, respectively. The centers of rectangles are placed at (−0.09 m, 0.3 m, 0 m), (0 m, 0.4 m, 0 m), and (0.09 m, 0.5 m, 0 m) from left to right, respectively. The reflection coefficient of the object under test is 1.

**Figure 7:**
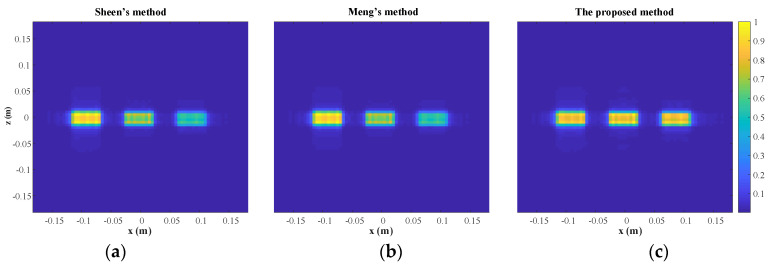
The reconstruction results. (**a**) Sheen’s method. (**b**) Meng’s method. (**c**) The proposed method.

**Figure 8:**
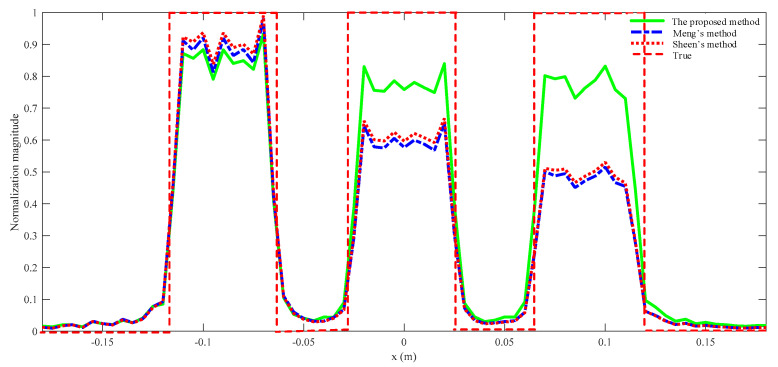
The reconstructed and true normalized magnitude along the line *z* = 0 m.

The rectangles are reconstructed by Sheen’s method, Meng’s method, and the proposed method. The normalized results are projected to *xz* plane as shown in Figure 7. All three rectangles are well reconstructed by the three methods. From a visual point of view, Figure 7a,b are quite similar, while Figure 7c is better than the other two. More specifically, the reconstructed rectangles centered at *y* = 0.3 m are similar across all three methods, but the reconstructed rectangles centered at *y* = 0.4 m and *y* = 0.5 m are better for the proposed method than for the other two methods. The magnitude along the line *z* = 0 m is plotted in Figure 8. It is evident that the reconstructed profiles of the two rectangles on the right by the proposed method are closer to the true profiles, which means that the proposed method can better focus when there are multiple objects within different ranges.

In order to evaluate the algorithms’ performance fairly, the same quantitative performance indicators, namely, the root mean square error (RMSE) and the structural similarity index measure (SSIM), are selected as evaluation indexes. The RMSE is defined as

(11) $$\mathrm{RMSE}=\sqrt{\frac{1}{m}\sum_{i=1}^{m}{\left[f_{real}\left(i\right)-f_{cal}\left(i\right)\right]}^{2}}$$

where $f_{real}\left(i\right)$ and $f_{cal}\left(i\right)$ are the real and calculated values at the *i*-th pixel in the true and recovered image, respectively.

And the SSIM is evaluated as

(12) $$\mathrm{SSIM}={\left[l\left(i\right)\right]}^{\alpha }\cdot {\left[c\left(i\right)\right]}^{\beta }\cdot {\left[s\left(i\right)\right]}^{\gamma }$$

where $l\left(i\right)$, $c\left(i\right)$, and $s\left(i\right)$ are the luminance comparison function, the contrast comparison function, and the structure comparison function, respectively [49]. In addition, α, β, and γ are 1 in this paper. When the recovered image is closer to the reference image, the SSIM is closer to 1 and RMSE is closer to 0.

The RMSE and SSIM are computed as shown in Table 2. Hence, it can be concluded that the more accurate physical model does yield a better performance of the proposed algorithm in terms of imaging quality, especially RMSE. Meanwhile, computational efficiency of the proposed algorithm drops only slightly as shown in Table 3. It is noted that there is a significant improvement in Figure 7 and Figure 8 but only very modest improvements in SSIM and RMSE. That is because the area of the object is a small proportion of the total imaging area.

### 3.3. The Full-Wave Data

**Figure 9:**
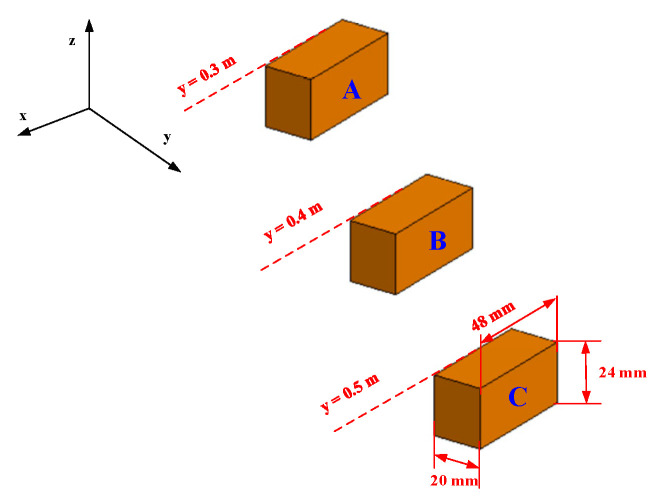
The CAD model of three blocks within different ranges.

In this section, the scattered electromagnetic field is computed by the commercial electromagnetic computation software FEKO. The CAD model of the target is presented in Figure 9. The target is assumed to be perfect electric conductors (PECs). The target is composed of three metal blocks of thickness 20 mm. The length and width of the block objects are 24 mm and 48 mm. The frontal center of the three block objects (namely A, B, and C) are located at (−0.09 m, 0.3 m, 0 m), (0 m, 0.4 m, 0 m), and (0.09 m, 0.5 m, 0 m), respectively.

**Figure 10:**
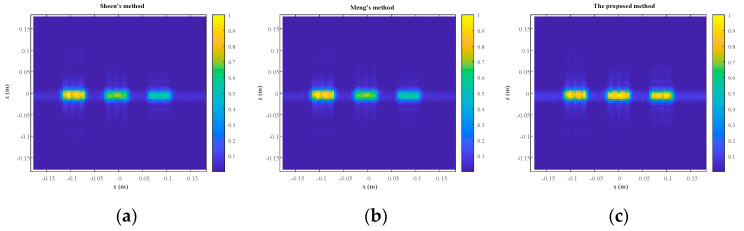
The front view of the reconstruction results. (**a**) Sheen’s method. (**b**) Meng’s method. (**c**) The proposed method.

**Figure 11:**
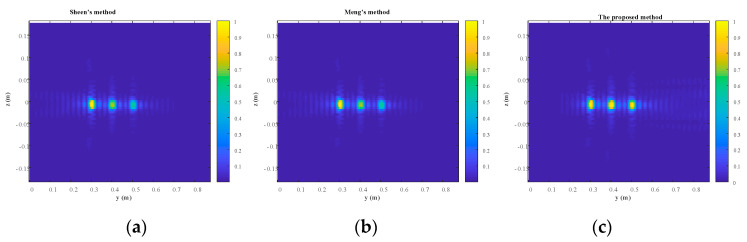
The side view of the reconstruction results. (**a**) Sheen’s method. (**b**) Meng’s method. (**c**) The proposed method.

**Figure 12:**
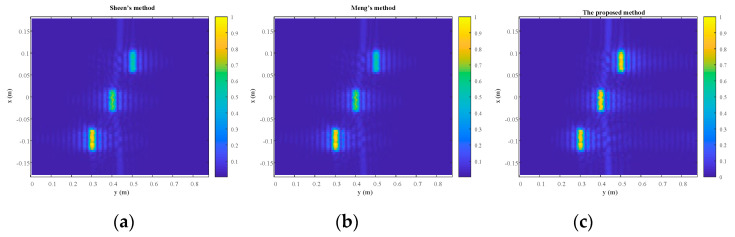
The top view of the reconstruction results. (**a**) Sheen’s method. (**b**) Meng’s method. (**c**) The proposed method.

**Figure 13:**
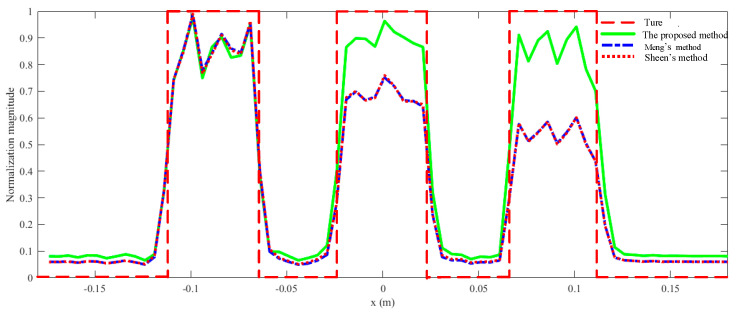
Profiles along *z* = 0 m in Figure 10.

**Figure 14:**
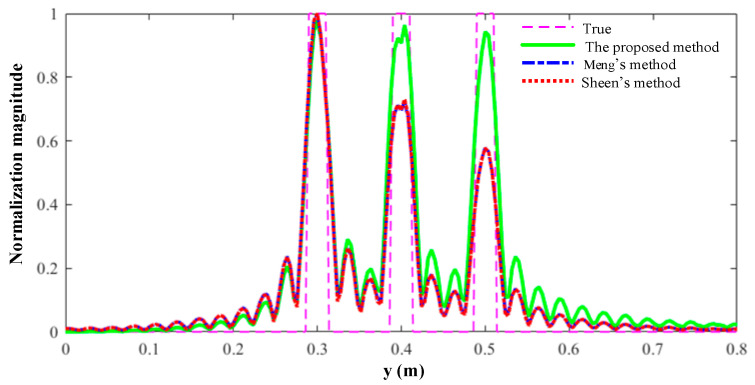
Profiles along *z* = 0 m in Figure 11.

The front, side, and top views of the reconstruction results are shown in Figure 10, Figure 11 and Figure 12, respectively. Overall, the quality of reconstruction results by the proposed algorithm is better than those by the other two methods. Specific to each block, block A is the best reconstructed of the three blocks by all three methods since it is closest to the transceiver plane. The reconstruction results of block A are almost the same across all three methods. For blocks B and C, it is evident that the reconstruction results by the competing methods become worse with the increasing distance between the blocks and the transceiver plane. However, the proposed method is less sensitive to distance, so its reconstruction results for blocks B and C are much better than those of the other two methods. Furthermore, the normalized magnitude along *z* = 0 in Figure 10 and Figure 11 is plotted in Figure 13 and Figure 14. Likewise insensitivity to distance, and accordingly better reconstruction results as shown in Figure 13 and Figure 14, are also very easy to observe. The proposed method better reconstructed the farther parts than did the other two methods. The SSIM and RMSE values given in Table 4 further confirm the superior performance of the proposed algorithm against its competitors. Once again, the proposed algorithm only suffers very little deterioration in computational efficiency as shown in Table 5.

### 3.4. Experimental Results

A millimeter-wave imaging prototype was built in the laboratory, which can collect real data to test in-house-developed imaging algorithms. There are 157 equivalent sampling points with the same spacing of 5 mm along the horizontal direction (*x*-direction) and 397 equivalent sampling points with 5 mm steps along the vertical direction (*z*-direction) in the imaging area of 0.8 m × 2 m. The operation frequency ranges from 27 GHz to 32.8 GHz with 220 equidistant sampling points. The acquisition time is only 2 s. The full details of the prototype in the laboratory can be found in [25].

**Figure 15:**
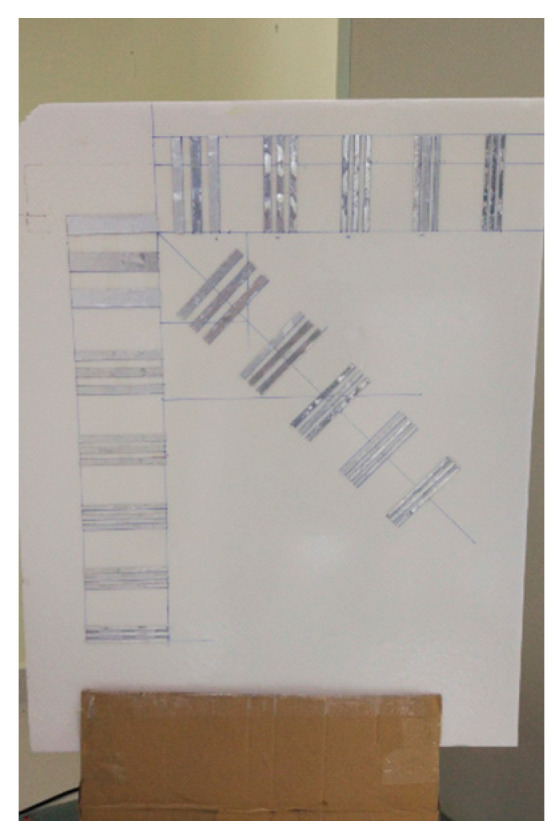
Photograph of the target under test.

The target contains three groups of perfectly conducting strips of length 100 mm as shown in Figure 15 Both the horizontal and diagonal groups have five sets of strips. The space between neighboring sets is 50 mm. The widths of strips in each set are 10 mm, 7 mm, 6 mm, 5 mm, and 4 mm, and the space between any two neighboring strips in the same set is the same as the strip width. However, the vertical group involves one more set of strips of 20 mm width and spacing. The test target is positioned in front of the transceiver plane center at a distance of 0.4 m.

**Figure 16:**
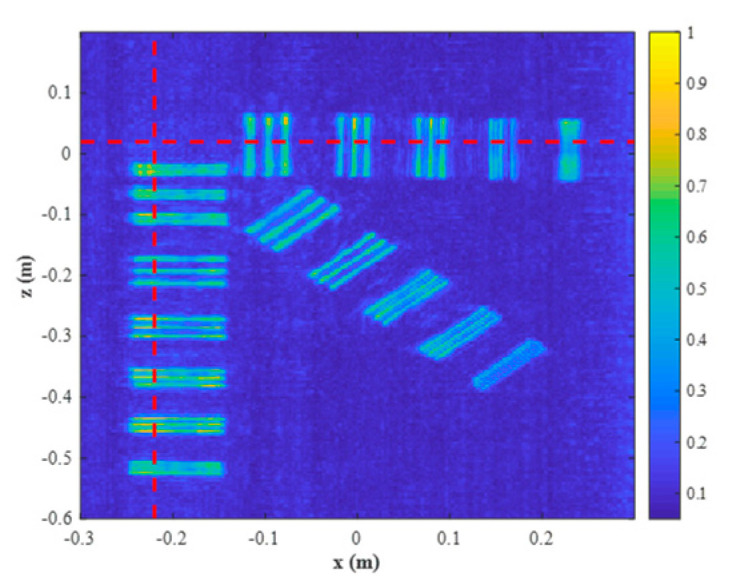
Zoomed-in reconstructed result.

**Figure 17:**
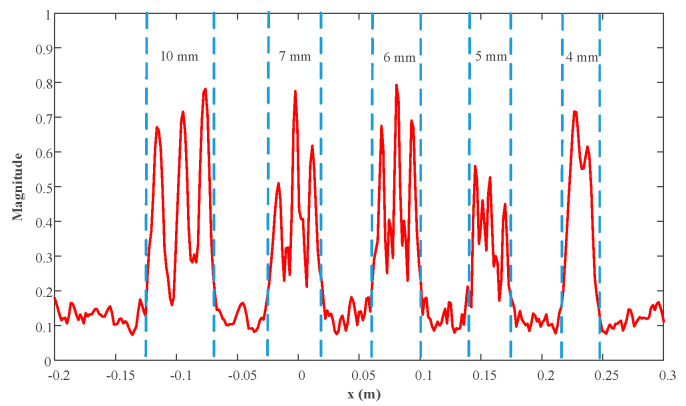
Profiles along the red horizontal line in Figure 16.

**Figure 18:**
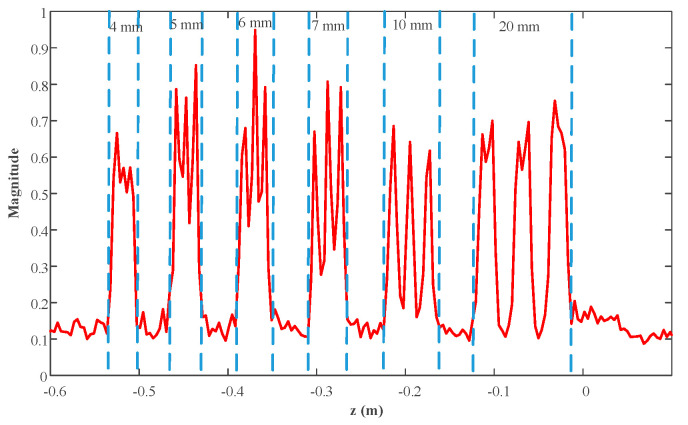
Profiles along the red vertical line in Figure 16.

The reconstruction result is given in Figure 16, which shows only the object under test for better visualization. The profiles along the red lines in Figure 16 are plotted in Figure 17 and Figure 18. It is evident that the strips of 5 mm and greater can be easily distinguished. Hence, the actual resolution of the proposed algorithm for the prototype is about 5 mm.

**Figure 19:**
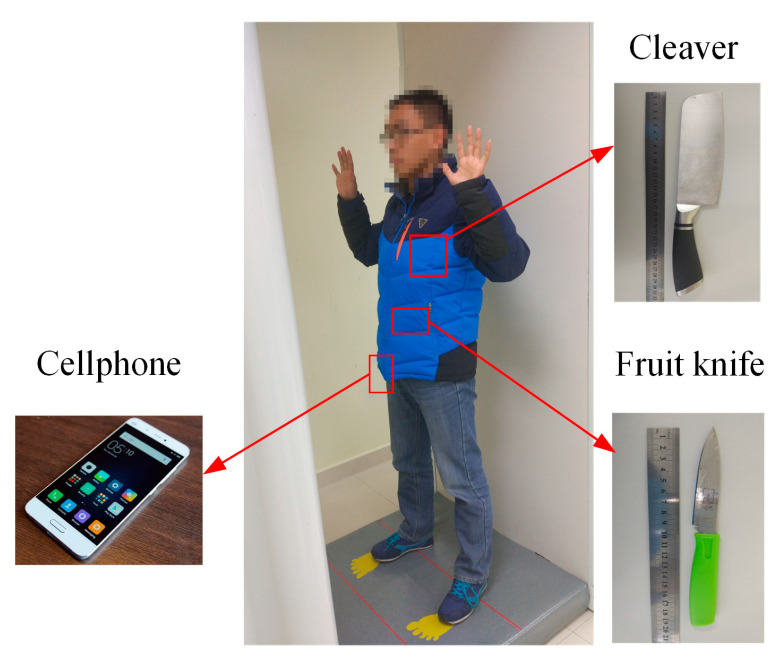
The scenario with a volunteer scanned by prototype.

**Figure 20:**
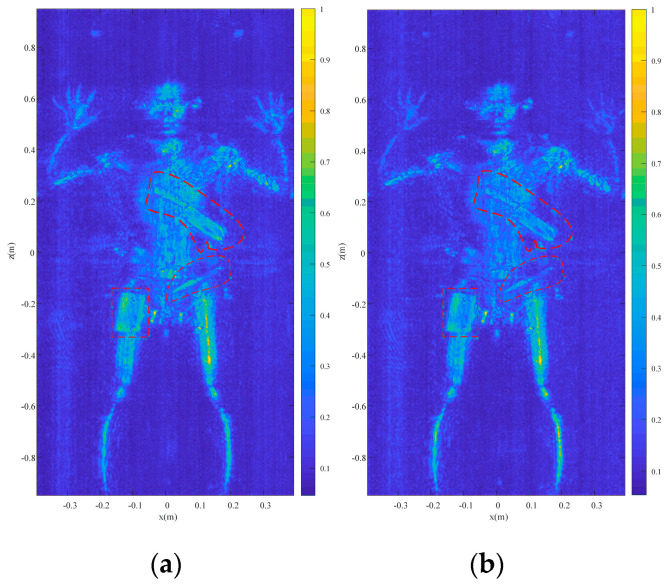
The reconstruction results (**a**) by Meng’s method and (**b**) by the proposed method.

Finally, another more complex real scenario is presented. A person who carried a cleaver, a fruit knife, and a cellphone, all hidden under a down jacket, was scanned by the prototype as shown in Figure 19. The distance between the volunteer and the antenna plane is also 0.4 m. The reconstruction results are given in Figure 20, which are marked from the circles and squares. All the concealed objects are detected and identifiably presented in the reconstructed images. The SSIM between the reconstruction results by Meng’s method and the proposed method is 0.8852.

## 4. Conclusions

In this paper, an electromagnetic imaging algorithm for a close-range monostatic system with dual path propagation loss has been presented for use in personnel screening for security applications. In this application, the scale of objects under scrutiny is comparable to the range of distances. The physical model treats incident waves and scattered waves as spherical waves with more rigorous amplitude term as per electromagnetic theory. As a consequence, the mathematical method in classical algorithms, such as spherical wave decomposition and Weyl identity, cannot handle the mathematical model derived from the physical model. The proposed algorithm is derived through MSP and is more accurate than the algorithms without dual path propagation loss. Consequently, the proposed method can achieve a better focusing effect for multiple targets in different range planes.

To verify the proposed algorithm, simulations and experiments have been carried out. PSF quantifies the algorithm resolution to be approximately 5.94 mm in the *x* and *z* direction and 24.3 mm in the range direction. Although the proposed algorithm shows slightly worse computing efficiency compared with Meng’s method and Sheen’s method, it outperforms its competitors in terms of quantitative indicators. Good performance has been observed by FEKO-simulated full-wave data from a three-dimensional target. The farther parts of the object were better reconstructed by the proposed method than by its competitors. Finally, millimeter-wave imaging using real data collected from our in-house-developed prototype demonstrated that the proposed algorithm performs well, as expected.

## Figures and Tables

**Table 1:** Simulation parameters adopted in synthetic system.

Parameters	Value
Center frequency	29.9 GHz
Frequency bandwidth	5.8 GHz
The number of sampling frequency points	220
The length of the aperture along x axis	360 mm
The sampling interval along x axis	5 mm
The length of the aperture along z axis	360 mm
The sampling interval along z axis	5 mm

**Table 2:** Comparison of SSIM and RMSE for synthesis data.

	Sheen’s Method [11]	Meng’s Method [25]	The Proposed Method
SSIM	9.495 × 10^−1^	9.501 × 10^−1^	9.544 × 10^−1^
RMSE	5.957 × 10^−3^	5.921 × 10^−3^	4.316 × 10^−3^

**Table 3:** Computation time for synthesis data.

Method	Time (s)
Sheen’s method	3.44
Meng’s method	3.54
The proposed method	3.61

**Table 4:** Comparison of SSIM and RMSE.

	Sheen’s Method [11]	Meng’s Method [25]	The Proposed Method
SSIM	9.2118 × 10^−1^	9.218 × 10^−1^	9.270 × 10^−1^
RMSE	1.0753 × 10^−3^	1.0714 × 10^−3^	8.774 × 10^−3^

**Table 5:** Computation time for simulation.

Algorithm	Time (s)
Sheen’s method	2.86
Meng’s method	2.93
The proposed method	2.94

## Data Availability

Not applicable.

## Appendix A

Here, the derivation of (6) by using MSP [36,41,45] is presented, which cannot be solved by the mathematical methods in classical algorithms, such as spherical wave decomposition and Weyl identity [11,25]. Let

(A1) $$\begin{array}{ll} E\left(k_{x},k_{z},k\right) & =\int_{-\infty }^{\infty }\int_{-\infty }^{\infty }\frac{\exp\left(-j2kR\right)}{R^{2}}e^{-jk_{x}x^{\prime }}e^{-jk_{z}z^{\prime }}dx^{\prime }dz^{\prime }\\ & =\int_{-\infty }^{\infty }\int_{-\infty }^{\infty }D\left(x^{\prime },y^{\prime }\right)\cdot e^{j2k\Phi \left(x^{\prime },z^{\prime }\right)}dx^{\prime }dz^{\prime } \end{array}$$

where

(A2) $$D\left(x^{\prime },z^{\prime }\right)=\frac{1}{{\left(x^{\prime }-x\right)}^{2}+{\left(y^{\prime }-y\right)}^{2}+{\left(z^{\prime }-z\right)}^{2}}$$

(A3) $$\begin{array}{ll} \Phi \left(x^{\prime },z^{\prime }\right)= & -\sqrt{{\left(x^{\prime }-x\right)}^{2}+{\left(y^{\prime }-y\right)}^{2}+{\left(z^{\prime }-z\right)}^{2}}\\ & -\frac{k_{x}}{2k}x^{\prime }-\frac{k_{z}}{2k}z^{\prime } \end{array}$$

When *k* is large and $\Phi \left(x^{\prime },z^{\prime }\right)$ reaches extreme value at a stationary point $\left(x_{s},z_{s}\right)$ in the integrating range, the major contribution to the integral in (A1) comes from the integral value at the neighborhood near stationary point $\left(x_{s},z_{s}\right)$.

At the stationary point, we have

(A4) $${\left.\frac{\partial \Phi }{\partial x^{\prime }}\right|}_{x_{s},z_{s}}=\frac{-\left(x_{s}-x\right)}{\sqrt{{\left(x_{s}-x\right)}^{2}+{\left(y^{\prime }-y\right)}^{2}+{\left(z_{s}-z\right)}^{2}}}-\frac{k_{x}}{2k}=0$$

(A5) $${\left.\frac{\partial \Phi }{\partial z^{\prime }}\right|}_{x_{s},z_{s}}=\frac{-\left(z_{s}-z\right)}{\sqrt{{\left(x_{s}-x\right)}^{2}+{\left(y^{\prime }-y\right)}^{2}+{\left(z_{s}-z\right)}^{2}}}-\frac{k_{z}}{2k}=0$$

The stationary point can be solved as

(A6) $$x_{s}=x-\frac{k_{x}}{\sqrt{{\left(2k\right)}^{2}-k_{x}^{2}-k_{z}^{2}}}\left(y^{\prime }-y\right)$$

(A7) $$z_{s}=z-\frac{k_{z}}{\sqrt{{\left(2k\right)}^{2}-k_{x}^{2}-k_{z}^{2}}}\left(y^{\prime }-y\right)$$

Here, the negative roots are extracted for each case, and it has been assumed that $y^{\prime }-y<0$.

The asymptotic approximation to (A1) is therefore

(A8) $$\begin{array}{ll} E\left(k_{x},k_{z},k\right) & =D\left(x_{s},z_{s}\right)e^{j2k\Phi \left(x_{s},z_{s}\right)}\\ & \;\cdot \int_{-\infty }^{\infty }\int_{-\infty }^{\infty }e^{\frac{1}{2}j2k\left[\alpha {\left(x_{s}-x\right)}^{2}+\beta {\left(z_{s}-z\right)}^{2}+2\gamma \left(x_{s}-x\right)\left(z_{s}-z\right)\right]}dxdz\\ & =\frac{2\pi j\sigma }{\sqrt{\left|\alpha \beta -\gamma^{2}\right|}}D\left(x_{s},z_{s}\right)\frac{e^{j2k\Phi \left(x_{s},z_{s}\right)}}{2k} \end{array}$$

where

(A9) $$D\left(x_{s},z_{s}\right)=\frac{{\left(2k\right)}^{2}-k_{x}^{2}-k_{z}^{2}}{{\left(2k\right)}^{2}{\left(y-y^{\prime }\right)}^{2}}$$

(A10) $$\alpha ={\left.\frac{\partial^{2}\Phi }{\partial {x^{\prime }}^{2}}\right|}_{x_{s},z_{s}}=-\frac{{\left(2k\right)}^{2}-k_{x}^{2}}{y^{\prime }-y}\cdot \frac{\sqrt{{\left(2k\right)}^{2}-k_{x}^{2}-k_{z}^{2}}}{{\left(2k\right)}^{3}}>0$$

(A11) $${\left.\beta =\frac{\partial^{2}\Phi }{\partial {z^{\prime }}^{2}}\right|}_{x_{s},z_{s}}=-\frac{{\left(2k\right)}^{2}-k_{z}^{2}}{y^{\prime }-y}\cdot \frac{\sqrt{{\left(2k\right)}^{2}-k_{x}^{2}-k_{z}^{2}}}{{\left(2k\right)}^{3}}$$

(A12) $${\left.\gamma =\frac{\partial^{2}\Phi }{\partial x^{\prime }\partial z^{\prime }}\right|}_{x_{s},z_{s}}=\frac{k_{x}k_{z}\sqrt{{\left(2k\right)}^{2}-k_{x}^{2}-k_{z}^{2}}}{\left(y^{\prime }-y\right)\cdot {\left(2k\right)}^{3}}$$

(A13) $$\sigma =\left\{\begin{array}{l} +1\;\mathrm{for}\;\alpha \beta >\gamma^{2},\;\alpha >0\\ -1\;\mathrm{for}\;\alpha \beta >\gamma^{2},\;\alpha <0\\ -j\;\mathrm{for}\;\alpha \beta <\gamma^{2} \end{array}\right.$$

(A14) $$\Phi \left(x_{s},z_{s}\right)=-\frac{\sqrt{{\left(2k\right)}^{2}-k_{x}^{2}-k_{z}^{2}}}{2k}\left(y-y^{\prime }\right)-\frac{k_{x}}{2k}x-\frac{k_{z}}{2k}z$$

Eventually, substituting (A9)–(A14) into (A8), the asymptotic approximation expression is

(A15) $$E\left(k_{x},k_{z},k\right)=\frac{j\pi }{k}\cdot \frac{1}{y-y^{\prime }}\cdot e^{-j\left(\sqrt{{\left(2k\right)}^{2}-k_{x}^{2}-k_{z}^{2}}\left(y-y^{\prime }\right)+k_{x}x+k_{z}z\right)}$$
